# Comparative breast cancer research, lessons from companion animals

**DOI:** 10.1186/1753-6561-7-S2-K9

**Published:** 2013-04-04

**Authors:** Jan A Mol

**Affiliations:** 1 Department of Clinical Sciences of Companion Animals, Faculty of Veterinary Medicine, Utrecht University, the Netherlands

## 

Breast cancer is the most frequent occurring tumor in humans. Current knowledge describes breast cancer as a heterogeneous disease classified in four major categories, i.e. basal-like, ErbB2 enriched, luminal subtype A or luminal subtype B. The majority of human breast cancers are hormone-receptor positive and may respond to endocrine therapy.

Our understanding of mammary gland development and breast cancer progression and metastasis comes mainly from cell lines and (transgenic) mouse models. However, these models have limitations. Differences exist between human and mouse tumorigenesis and metastasis. Spontaneous mouse models in general also do not present hormone-sensitive mammary cancers. The available chemically- or virally-induced mouse models or genetically manipulated mouse models are also more homogeneous and represent only certain phenotypes of the human disease.

Spontaneous mammary carcinomas in companion animals may present as relevant to be studied both for veterinary treatment and increase in knowledge and treatment modalities of human breast cancer. Companion animals are exposed in a similar way to humans to environmental mutagens or carcinogens in food. The annual incidence of mammary carcinomas in the dog is 2 to 3-fold higher than in humans whereas a 4-fold lower incidence of mammary cancer is found in the cat. The high incidence of spontaneous mammary cancer in dogs and the higher homology of the canine genome to the human genome than that of rodents make them relevant for human breast cancer research. Comparison of gene expression profiles of mammary cancer in humans and dogs showed that they share similar pathways involved in tumorigenesis such as integrin-signalling, Wnt-signalling, chemokine and cytokine signalling and angiogenesis. Also perturbations in cancer-related pathways such as PI3K/AKT, PTEN and MAPK are found. Immunohistochemical analysis of canine mammary tumors identifies the luminal A and B and basal-like tumors as found in humans. Part of the canine mammary tumors is hormone receptor positive although the presence of progesterone- and estrogen-receptors decreases with increasing malignancy.

Studies at the start of this century in women receiving hormone-replacement therapy (HRT) showed an increased incidence of breast cancer in the group receiving a combination of estrogens and progestins and not in the estrogen-only group resulting in a paradigm shift in the thinking about the role of progesterone in breast cancer development. In dogs and cats progesterone plays a dominant role in mammary tumorigenesis. We will therefore focus on the role of progesterone.

The signal transduction cascades involved in progesterone signaling start with binding of progesterone to specific receptors. The two well-known nuclear progesterone receptors (PR) are transcribed from a single gene but - through use of different promoters - two different PR isoforms are synthesized. The shorter form, PRA, contains the hormone binding domain, a hinge region and a DNA binding domain but lacks an amino-terminal sequence which is unique for the longer PRB receptor. This B-upstream region segment (BUS) contains an activation domain, AF3, which results overall in a much higher transactivation potential of the PRB in relation to PRA. PRA is mainly found in nuclei whereas PRB has both nuclear and cytoplasmic localizations. Progesterone plays a central role in the regulation of stem and progenitor cells within the mammary gland. Cells expressing the PR act as sensors and upon progesterone binding they stimulate the stem cell compartment to proliferate and differentiate.

In the cat, endogenous progesterone and synthetic progestins induce fibroadenomatous hyperplasia (FAH) of the mammary gland. In such FAH tissues we demonstrated both PR isoforms PRA (80-86 kDa) and PRB (116-120 kDa) using Western blots. In FAH examined by immunohistochemistry we found predominant staining for PR in ductal epithelium with both nuclear and cytoplasmic localization. Compared to feline mammary carcinoma PR expression as measured by Q-PCR was greatly elevated in FAH with a concomitant increased expression of growth hormone (GH), GHR and IGF-I mRNA. Cats with FAH can be treated successfully with a PR antagonist.

In the dog exposure to endogenous progesterone or to synthetic medroxyprogesterone acetate (MPA) may result in acromegalic changes and mammary hyperplasia. Expression analysis revealed induction by MPA of the expression of GH and IGF-I mRNA in the mammary gland, whereas GHR and PR expression were down-regulated. Gene-profiling studies revealed induction of the Wnt-pathway. Immunohistochemical analysis of the PR revealed definite nuclear and cytoplasmic staining in mammary epithelium. Staining for PR was positive in epithelial cells in proliferative zones of the mammary gland whereas in differentiated mammary tissue PR-positive cells were low. No staining was found in stromal or myoepithelial cells. In mammary tumors marked heterogeneous staining was found including perinuclear staining of tumorous cells and cytoplasmic staining in spindle cells. In addition, all GH-producing cells were positive for PR emphasizing the relation between progesterone exposure and local mammary GH expression.

Recently we studied the transactivation potential of the canine PRA and PRB isoforms. Human and canine PRA had a comparable transactivation potential, but in vitro studies revealed a lower and limited transactivation potential of the canine PRB in comparison to human PRB. Interestingly human breast cancer with a high PRA:PRB ratio are associated with a higher malignant phenotype.

In conclusion, progesterone plays an important role in mammary hyperplasia and carcinogenesis in companion animals inducing among others Wnt- and cytokine signaling thereby stimulating recruitment of mammary stem cells. The spontaneous nature and high incidences make them attractive for further studies on the hormonal regulation of breast cancer development and treatment.

## Competing interests

There are no competing interests in this presentation.

